# Using intervention mapping to develop an outpatient nursing nutritional intervention to improve nutritional status in undernourished patients planned for surgery

**DOI:** 10.1186/s12913-020-4964-6

**Published:** 2020-02-27

**Authors:** Harm H. J. van Noort, Maud Heinen, Monique van Asseldonk, Roelof G. A. Ettema, Hester Vermeulen, Getty Huisman-de Waal, On the behalf of the Basic Care Revisited (BCR) Research group, J. P. H. Hamers

**Affiliations:** 1 Department of Nutrition, Physical Activity and Sports, Department of Surgery, Gelderse Vallei Hospital, P.O. Box 9025, 6710 HN Ede, The Netherlands; 2 Radboud University Medical Centre, Radboud Institute for Health Sciences, IQ Healthcare, P.O. Box 9101, 6500 HB Nijmegen, The Netherlands; 3 Department of Gastroenterology and Hepatology - Dietetics and Intestinal Failure, Radboud University Medical Center, Nijmegen, The Netherlands; 4 Julius Center for Health Sciences and Primary Care, University Utrecht Str. 6.131, P.O. Box 85500, 3508 GA Utrecht, The Netherlands; 5 Research Center Health and Sustainable Living, Utrecht University of Applied Sciences, P.O. Box 12011, 3501 AA Utrecht, The Netherlands; 6 Faculty of Health and Social Studies, HAN University of Applied Sciences, Nijmegen, The Netherlands

**Keywords:** Nursing, Undernutrition, Nutritional support, Preoperative care, Needs assessment, Health behaviour change, Development, Intervention mapping, Outpatient clinic, Prehabilitation

## Abstract

**Background:**

Undernutrition in surgical patients leads to a higher risk of postoperative complications like infections and delayed recovery of gastrointestinal functions, often resulting in a longer hospital stay and lower quality of life. Nurses at outpatient clinics can deliver nutritional care during outpatient preoperative evaluation of health status to ensure that patients are properly fed in preparation for hospital admission for surgery. However, nutritional nursing care was not determined in research yet. This paper describes the structural development of an Outpatient Nursing Nutritional Intervention (ONNI).

**Methods:**

A project group followed the steps of the Intervention Mapping. The needs assessment included assessment of delivery of nutritional care and nutritional care needs at two anaesthesia outpatient clinics of an academic and a teaching hospital. Also, outpatient clinic nurses and patients at risk for undernutrition were interviewed. Determinants resulted from these methods were matched with theories on behaviour change and nutritional support.

**Results:**

Both patients and nurses were unaware of the consequences of undernutrition, and nurses were also unaware of their roles with regard to nutritional support. The intervention goals were: 1) enabling surgical patients to improve or maintain their nutritional status before hospital admission for surgery, and 2) enabling nurses to deliver nutritional support. The ONNI was developed for outpatients at risk for or with undernutrition. A training was developed for nurses. The ONNI included the five following components: 1) identification of the causes of undernutrition; 2) provision of a nutritional care plan including general and individually tailored advice; 3) self-monitoring of nutrient intake; 4) counselling and encouragement; and 5) support during a telephone follow-up meeting. The intervention and training were tested. A multifaceted implementation strategy was used to deliver the intervention in daily practice.

**Conclusions:**

Despite the unique position of the nurses at outpatient clinics, nurses were unaware of their role with regard to nutritional care. The ONNI was developed and implemented along with a training program for nurses. The test confirmed that the training can improve nurses’ knowledge, skills, and sense of responsibility for nutritional support. The intervention may empower patients to actively improve their nutritional status.

## Background

Undernutrition is an important prognostic indicator of postoperative complications, such as infections, fistulas or wound-healing problems, and the delayed recovery of gastrointestinal functions [1, 2]. Additionally, undernourished surgical patients face more renal and cardiac complications [1], and prolonged hospital stays [3]. Undernutrition can be measured timely with screening instruments such as the Malnutrition Universal Screening Tool (MUST) [4] and Short Nutritional Assessment Questionnaire (SNAQ) [5]. With these instruments, undernutrition was found among 14% of 564,063 patients admitted to Dutch hospitals [3]. An Italian study at medical and surgical units found that 18% (*n* = 60) and 45% (*n* = 155) of surgical inpatients were undernourished or at risk for undernutrition [6]. In a sample of gastrointestinal surgical patients in a university hospital in the USA, 19% (*n* = 93) were moderately or severely undernourished based on screening at the time of admission [2]. In The Netherlands, preoperative assessment of nutritional status using SNAQ at outpatient clinics demonstrated that 5% (*n* = 49) to 7% (*n* = 67) of surgical patients were moderately to severely undernourished [5, 7]. These studies in especially high-income countries signify higher undernutrition rates for surgical inpatients as compared to outpatients. This suggests that undernutrition in surgical patients worsens in the period between outpatient clinic visit and hospital admission. Thus, it is pivotal that patients’ nutritional status should be improved as early as possible to benefit their outcomes.

To ensure that surgical patients are properly fed, nutritional prehabilitation is needed. Studies on nutritional support before and after surgery have demonstrated positive effects on infections and length of hospital stay [8, 9]. Nutritional support, or nutritional therapy, is defined by the European Society for Clinical Nutrition and Metabolism (ESPEN) as the provision of nutrition – either orally (including regular or therapeutic diet and oral nutritional supplements (ONS)), through enteral (EN) administration, or parenteral (PN) administration [10]. The meta-analysis of RCTs by Zhong [8] and Burden’s Cochrane review [9] illustrated these effects, through ONS, EN, and PN methods at different periods before, during, and after surgery. Studies evaluating oral nutritional support using regular or therapeutic diet preoperatively were identified in our systematic review and demonstrated improved nutritional status or prevention of further decline of undernutrition [11]. The intervention components determined in our systematic review study were education, monitoring of dietary intake, individually tailored advice regarding symptoms, and follow-up. However, only a small number of intervention studies were found (*n* = 5).

In The Netherlands, surgical patients’ health status including nutritional screening is evaluated before surgery by both nurses and anaesthesiologists during outpatient preoperative evaluations [5, 7, 12]. In this setting of health care service, nurses are in key positions to provide nutritional support to improve or maintain patients’ nutritional status. Systematic reviews of nutritional nursing did not, however, identify intervention studies in which nurses provided oral nutritional support preoperatively during outpatient clinic consults [11, 13]. A nutritional supportive intervention to be delivered by nurses should be developed for use in outpatient clinic services for pre-operative health evaluation to prehabilitate undernourished surgical patients.

### Intervention development

Nutritional prehabilitation of surgical patients can be considered a complex intervention. Complex interventions are the focus of the Medical Research Council (MRC) framework, which provides guidance for development and evaluation [14]. Complex interventions encompass several interacting components, numerous and varied outcomes, several behaviours to deliver or receive the intervention, different target groups, and the need for flexibility or tailoring [14]. Regarding nutritional prehabilitation, the need of tailoring varies based on the different causes of disease-related undernutrition and amount of time before surgery. Healthcare professionals face different groups of patients based on different classes of nutritional risk, e.g., low risk (well-nourished), medium risk (at risk for undernutrition), or high risk (undernutrition) [4]. Furthermore, both patients and outpatient clinic nurses have to change behaviour routines [15]. Therefore, development of the complex preoperative nutritional optimization requires a systematic approach [14].

**Fig. 1:**
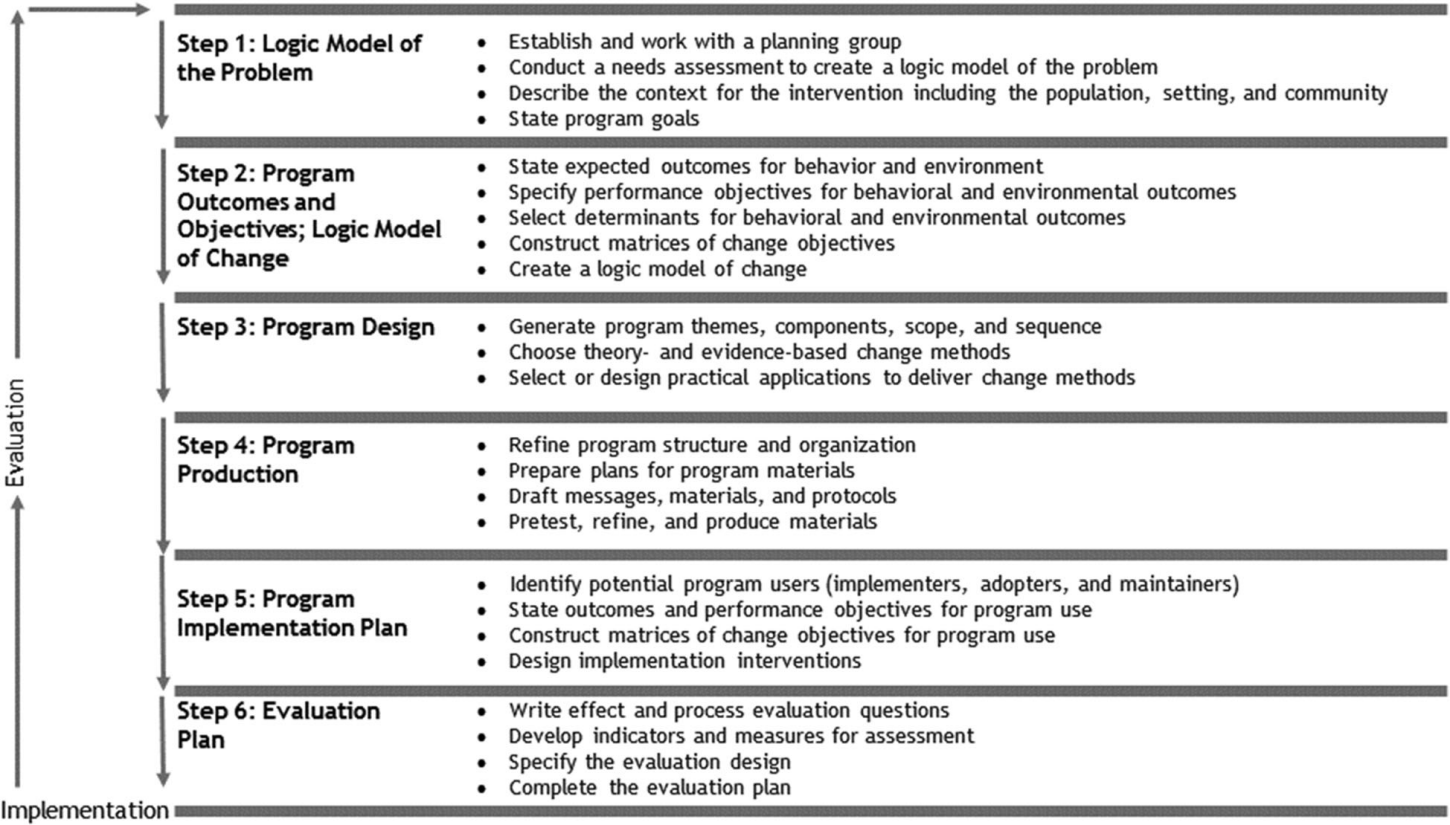
The six steps of Intervention Mapping

Systematic intervention development of new interventions is defined by Bartolomew and colleagues in the Intervention Mapping (IM) approach [16]. Intervention Mapping is a framework [17] that includes a systematic, iterative six-step process, which helps researches and healthcare professionals to develop or adapt an intervention based on theoretical, empirical, and practical information [16]. This framework has been used widely for health promotion, e.g., nutrition [18, 19], as well as in other basic nursing care programs [20, 21]. The steps in IM are as follows: 1) Logic Model of the Problem; 2) Program outcomes and Objectives – Logic Model of Change; 3) Program Design; 4) Program Production; 5) Program implementation plan; and 6) Evaluation plan [16] (see Fig. 1 and Table 1). Each step encompasses clear tasks and a clear end product. We used the IM to structure the development of an Outpatient Nursing Nutritional Intervention (ONNI). In this paper we describe the methods that are used during the development and the end products that were developed. The methods and results are presented for each step are presented separately. This development is part of the Basic Care Revisited Research program [28].

**Table 1:** Methods used to develop and implement ONNI^a^ following the six steps of Intervention Mapping

Six steps of Intervention Mapping	Study objectives	Methods used during the development
Step 1: Logic Model of the Problem		
Aim: to examine a specific health problem in the target population	To examine the behavioural and environmental determinants of undernourished patients planned for surgery seen at anaesthetic outpatients clinics	• Interviews with patients and nurses, observations of nutritional care, survey among patients (see Table 2)
Step 2: Program Outcomes and Objectives – Logic Model of Change		
Aim: to develop matrices of change objectives	To define program outcomes, performance objectives, change objectives	• Panel discussion and definition session • Matrix of program objectives (see Tables 3 & 4)
Step 3: Program Design		
Aim: to generate program ideas, including change methods and practical applications	To generate program idea’s with methods for change	• Theory of undernutrition and nutritional support • Theory of behaviour change • Implementation strategies (see Table 5)
Step 4: Program Production		
Aim: to produce a programme that matches the previous steps	To produce a program for undernourished patients during outpatient preoperative evaluation at anaesthetic outpatient clinic	• Development of the ONNI^a^ (see Table 6) • Development of a nursing nutritional training • Pre-test of the ONNI^a^ and the training
Step 5: Implementation Plan		
Aim: to develop an implementation plan to enable adoption, implementation, and maintenance	To develop an implementation plan of the ONNI^a^	• Identification of implementation barriers and process evaluation (see Table 7) • Literature on implementation strategies and evaluation of complex interventions

^a^Outpatient Nursing Nutrition Intervention

## Step 1: logic model of the problem - methods

A project group was established to participate in the development of the intervention. The group was made up of a nurse, a nurse specialist, a dietician (MvA), a gastroenterologist, two researchers (GHdW, MH), and an external dietetic expert (HK). The specific context included two anaesthesia outpatient clinics for preoperative evaluation from a general and an academic hospital in the Netherlands. Nurses held consults with patients who were being seen mainly for general (e.g. vascular, abdominal), orthopaedic, neurological, plastic, or facial surgery. The nursing staff at the outpatient clinic from the academic hospital was made up of bachelor nurses. The nursing staff at the outpatient clinic from the general hospital was made up of bachelor nurses and nursing assistants. These settings were studied during the period between November 2014 and June 2016. The study was ethically approved by Medical Ethical Committee of the Radboud university medical centre in Nijmegen, The Netherlands, number 2014–1353.

Participants of all four studies were requested to provide written informed consent before participation. First, the behavioural and environmental determinants were uncovered through a needs assessment of the context in which the intervention would be performed. The needs assessment was conducted in four consecutive studies. Each study is described below and illustrated in Table 2.

**Table 2:** Studies conducted to determine the behavioural and environmental determinants (step 1)

Study	Aim	Sampling	Characteristics	N (%)
Nurses’ perspectives	To explore nurses’ perspectives towards nutritional care for undernourished surgical patients	Purposive sample of outpatient clinic nurses and nursing assistants (*N* = 10)	Nurses	
			Urology outpatient clinic	1 (10)
			Anaesthesia outpatient clinic	4 (40)
			Nursing assistants	5 (50)
Observation of nutritional care	To observe delivery of nutritional care during nursing consults at outpatient clinics	Consecutive consults (*N* = 341) at the anaesthesia outpatient clinic before a planned surgery in two hospitals	Academic Hospital	48 (14)
			General Hospital	293(86)
			Female	201 (59)
			Age (mean/SD)	55.3 (15)
			MUST^a^ score 0^b^	295 (88)
			MUST^a^ score 1	24 (7)
			MUST^a^ score 2	16 (5)
Survey	To evaluate patients’ satisfaction with general and nutritional care received during the outpatient clinic visit	Patients (*N* = 301) at anaesthesia outpatient clinic for preoperative screening	Female	156 (60)
			Age (mean/SD)	54 (16)
			MUST^a^ score 0^c^	236 (91)
			MUST^a^ score 1	14 (5)
			MUST^a^ score 2	9 (4)
Patients’ perspectives	To explore patients’ perspectives towards undernutrition and satisfaction with nutritional care	Patients (*N* = 11) from an academic hospital	Female	7 (64)
			Age (mean/SD)	55.7 (19.6)
			MUST^a^ score 1	8 (73)
			MUST^a^ score 2	3 (27)

^a^Malnutrition Universal Screening Tool; ^b^nutritional risk screening was performed in 335 (98.2%) of the 341 observed consults. ^c^surveys were returned by 259 (86%) of the patients

### Study 1: nurses’ perspectives

Semi-structured, face-to-face interviews with the nursing staff from both outpatient clinics were held by two 4th year students of Bachelor of Nursing to explore nurses’ perspectives towards nutritional care. The students worked under supervision of a senior researcher who coordinated the study and established the relations with the nursing staff of both outpatient clinics (GHdW). The complete nursing staff consisting of nurses and nursing assistants who evaluated health status before a planned surgery were selected and participated after recruitment by face-to-face contact and email. Interviews were based on the Integrated Change Model [29, 30]: a) awareness, b) self-efficacy and skills, c) attitude, and d) current care regarding (risk for) undernutrition (see Additional file 1). Interviews were held in separate rooms and were recorded on audio after informed consent was obtained. Audio records were transcribed and analysed using open coding in iterative discussion sessions of the two students and the researcher (GHdW). Then, a coding tree was built and codes were categorised based on determinants of the Integrated Change Model in a thematic analysis approach.

### Study 2: observation of nutritional care

Delivery of nutritional care according to the hospitals’ protocol was observed during nursing consults at the outpatient clinic. The protocol included a) screenings for undernutrition with MUST [4], and b) nutritional interventions for patients at risk for or with undernutrition. The MUST is a screening tool, made up of three independent criteria for protein - energy undernutrition and can result in a maximum total score of 6. A score of 0 indicates low risk (well-nourishment), score of 1 indicates medium risk (at risk for undernutrition), and a score of at least 2 indicates high risk (undernutrition). For a patient at risk for or with undernutrition, interventions should be performed. The nutritional interventions included the following: 1) provision of a leaflet with information about protein-rich food; 2) oral information about undernutrition, reasons for weight loss, and advice about protein-rich nutrition; and 3) referral to a dietician in case of MUST-scores ≥2. Protocol activities structured the observation list. Descriptive analyses were used to describe nurses’ adherence to the protocol.

### Study 3: survey

The Consumer Quality Index [31] was tailored to suit the outpatient setting in a survey (see Additional file 2) designed to evaluate patients’ satisfaction with the general and nutritional care received during the outpatient clinic visit. The main topics of the survey included a) the care received from the nursing staff, b) information needs regarding nutrition, and c) perspectives on personal nutritional status and general health status. Descriptive analyses were performed to describe the sample of patients and the results of the survey.

### Study 4: patients’ perspectives

Semi-structured interviews were held with patients of the academic hospital after the consult with the nurse at the outpatient clinic. These patients visited the clinic in preparation for surgery. Nurses contacted the researcher about patients at risk for or with undernutrition. These patients were recruited for the study by telephone. Under supervision of a senior researcher (GHdW), two 4th year students of Bachelor of Nursing students performed the interviews if patients provided informed consent. Based on the Integrated Change Model [29, 30], the topics selected were a) patients’ knowledge, attitudes, responsibilities, and motivations regarding undernutrition and nutritional intake, b) patients’ needs and expectations regarding nutritional care, and c) patients’ experiences with received nutritional care (see Additional file 3). The interview guide was pretested. Audio records were made and were transcribed and analysed through open coding and a thematic analysis using the determinants of the Integrated Change Model [29, 30].

## Step 1: logic model of the problem - results

Some clear determinants resulted from the needs assessment. First, nurses did not regularly discuss nutritional risk and did not give advice to undernourished patients. Moreover, nurses did not feel capable of providing nutritional support, and some nurses did not feel that it was their responsibility either. Patients were unaware of their nutritional status. If nutritional status was discussed, patients felt responsible and capable of taking care of their own nutritional intakes. Detailed determinants and results from the four studies follow below.

### Study 1: nurses’ perspectives

Ten nurses were interviewed, and five determinants to possibly influence nutritional care were derived from the analysis: current care, attitude, knowledge, skills and self-efficacy, and barriers.

Current care: some nurses complained that nutritional care only included screening of nutritional status. Most of the nurses complained that (under) nutrition was poorly discussed and that advice remained superficial and was provided unsystematically.

*‘I tell patients to ‘keep in mind to eat a varied diet’, but, I am not a food expert’.*

*(Nurse 4)*

Attitude: Some nurses regarded nutrition as their responsibility. Other respondents argued that dieticians are in leading positions with regard to nutrition on account of their expertise. Nurses themselves should signal nutritional problems, but nutritional advice and sufficient food intake of patients were not considered part of nursing. As such, these elements were not considered to be nurses’ responsibilities.

Knowledge, skills, and self-efficacy: Nurses did not uniformly deliver nutritional care, and some nurses did not know how to deliver nutritional care. The reasons cited were due to lack of time during the consults, lack of knowledge concerning undernutrition, and lack of adequate interventions. Nurses felt capable and familiar with screening for nutritional risk using MUST, but did not feel capable of advising undernourished patients about nutrition. All respondents expressed the need to be educated about their roles and (under)nutrition.

*‘I think that it is something that is added to our list, but we do not know what our role should be’.*

*(Nurse 3)*

Barriers: One of the barriers was a lack of privacy during the nursing consults in one of the hospitals because two patients are seen at the same time in one room. Therefore, nurses felt inhibited from discussing nutrition and nutritional status. Another barrier was that nurses were not giving nutrition a high priority, reflected in the fact that they said there is a lack of time. An additional barrier was inadequate weight measurement of patients in wheelchairs or with orthopaedic instruments.

### Study 2: observation of nutritional care

Nutritional status was screened in 98.2% (*N* = 335) of the patients, of whom 7% (*n* = 24) were found to be at increased nutritional risk and 5% (*n* = 16) were undernourished (see Table 2). Leaflets were provided to 75% (*n* = 30) of the patients. Only 10% (*n* = 4) of the patients received verbal information from the nurse. Referral to a dietician was arranged for 94% (*n* = 15) of the patients with undernutrition.

### Study 3: survey among patients

The survey was returned by 86% patients (*N* = 259) of which 228 (88%) provided answers on all questions. Risk for undernutrition and undernutrition were found in 5% (*n* = 14) and 4% (*n* = 9) of patients, respectively. The outpatient clinic’s overall care was valued at an 8.5 on scale from zero to 10 (0 indicating very poor care and 10 indicating ideal care). More than half of the patients (54%, *n* = 123) stated that they needed additional information regarding nutrition. Main information needs dealt with the following topics: a) adequate nutrition before surgery (34%, *n* = 77); b) energy and protein-rich food products (15%, *n* = 33); and c) organizing mealtimes during the day (8%,*n* = 19).

### Study 4: patients’ perspectives

Eleven patients were interviewed with an mean length of time for each interview of approximately 30 min. The analysis resulted in the following determinants: current care, awareness and attitude, knowledge, and skills and self-efficacy.

Current care: Most patients (*n* = 9) did not receive any nutritional advice during the consult at the outpatient clinic and did not have any expectations for outpatient clinic professionals with regard to nutritional care either.

*‘No, they did not mention anything [how to improve dietary intake]’.*

*(Patient 9)*

Patients who were referred to the dietician claimed that the advice was not applicable to their personal needs.

‘*The dietician handed me a whole list what I could eat during the day but that was way too much for me, that was not achievable’.*

*(Patient 7)*

Awareness and Attitude: Patients were unaware of their nutritional risk after screening at the outpatient clinic. Patients did not experience undernutrition as a problem for their health and recovery after surgery (see quotations).

*‘No, I don’t know about that, for me it was... yes, I was really surprised to hear that I am undernourished’.*

*(Patient 1)*

*‘This [being undernourished] sounds like a real problem, for me it is more like ...uh... weighing a little too less’.*

*(Patient 2)*

Adequately informed patients stated that they felt responsible for adequate nutritional intake.

Knowledge, skills, and self-efficacy: Patients did not know what undernutrition could mean for their recovery after surgery. They felt capable of eating a varied diet. Some patients stated that they do know what to do to maintain an adequate weight.

*‘Well, meanwhile I know the way to maintain weight’.*

*(Patient 7)*

Patients who received adequate information and advice stated that they were able to achieve adequate nutritional intake.

## Step 2: program outcomes and objectives – methods

The results of step 1 enabled the project group to define program goals for undernutrition and its behavioural and environmental causes. This was done by discussion panel with stakeholders. The stakeholder panel consisted of two patients, a nurse, a dietician (MvA), an external expert (HK) in clinical undernutrition, and two researchers (GHdW, MH). The discussion started with explaining the gap between the current situation and the ultimate goal that patients are in good nutritional condition before surgery. The current situation was explained by presenting the results of step 1. Then, the stakeholders discussed what should be accomplished to close this gap (program goals). This resulted in program goals and performance objectives. Also, they discussed which determinants needed to be changed. Then, the project group specified the performance objectives and linked these to the changeable determinants (step 1). By linking the performance objectives with the changeable determinants, the project group defined change objectives. Finally, researchers constructed a matrix of program goals, performance objectives, and relevant determinants for both patients and nurses.

## Step 2: program outcomes and objectives - methods

To close the gap between the current situation and a good nutritional condition before surgery the stakeholders and project group argued that behaviour change was needed in nurses as well as in patients. The programme goals for patients and nurses were as follows:

Patients at risk for or with undernutrition and planned for surgery maintain or improve their nutritional status.

Nursing staff at anaesthesia outpatient clinics support patients in achieving adequate nutritional intake, leading to maintenance or an improvement in patients’ nutritional status.

The goal for patients contains ‘improve’, in order to achieve the good nutritional condition. ‘Maintain’ was also mentioned in the goal in order to prevent further decline of undernutrition if improvement is too optimistic.

Matrices of both patients’ and nurses’ performance objectives, determinants, and change objectives were defined and are shown in Tables 3 and 4. Based on evidence from step 1, awareness and attitude, knowledge, skills, and self-efficacy were perceived as important and changeable determinants for patients’ performance objectives. These determinants are regarded as preconditions for improving nutritional status and were used to define the patients’ change objectives (Table 3). For nurses, the determinants knowledge, self-efficacy and skills, and attitude were perceived as important for the nurses’ performance objectives. By matching these (the determinants and performance objectives), the change objectives were defined (Table 4). We illustrate this matching for one performance objective in the next paragraph.

**Table 3:** Patients’ performance objectives, determinants and change objectives

Program goal: Outpatients at risk for or with undernutrition and planned for surgery are able to improve or maintain their nutritional status.				
Performance objectives	Important and changeable determinants and the related change objectives			
	Knowledge	Skills and Self-efficacy	Awareness and attitude	Outcome expectation
Patients are motivated to improve their nutritional status.	Patients understand their nutritional status. Patients have knowledge of the consequences of undernutrition regarding their health, treatment and recovery.	Patients demontrate to be capable and motivated to improve their nutritional status	Patients acknowledge the risk of undernutrition during their treatment course. Patients acknowledge the need to improve nutritional status to diminish the consequences of undernutrition.	Patients expect to become well-nourished before the planned surgery.
Patients take action regarding the personal cause (s) of undernutrition.	Patients know the cause (s) of undernutrition in their individual situation. Patients know how to diminish the cause’s of undernutrition.	Patients apply advices given to the personal cause (s) of undernutrition.	Patients explain causes of undernutrition for their individual situation. Patients are aware of the need to diminish the cause’s of undernutrition.	Patients expect to decrease the influence of the personal cause (s) of undernutrition.
Patients eat healthy, energy and protein enriched nutrition.	Patients have knowledge of healthy, energy and protein enriched nutrition.	Patients plan to buy, prepare and eat healthy, energy and protein enriched nutrition.	Patients are aware of the need to eat healthy, energy and protein enriched nutrition.	Patients expect to benefit from eating healthy, energy and protein enriched nutrition.
Patients have an adequate nutritional intake.	Patients have knowledge of their eating pattern. Patients know what they need to change regarding their eating pattern to have an adequate intake.	Patients demonstrate to change their eating pattern and to have an adequate nutritional intake.	Patients are aware of their eating pattern. Patients are aware of the need to change their eating pattern to have an adequate intake.	Patients expect to improve nutritional status by having an adequate nutritional intake.

**Table 4:** Nurses’ performance objectives, determinants and change objectives

Program goal: Nursing staff at anaesthesia outpatient clinics support patients in achieving an adequate nutritional intake, leading to an improvement or maintenance in patients’ nutritional status.				
Performance objectives	Important and changeable determinants and the related change objectives			
	Knowledge	Self-efficacy and skills	Attitude	Outcome expectation
Nurses offer patients the intervention map at the outpatient clinic.	K1.The nurse knows that the intervention map has to be offered to the patient during the outpatient clinic visit.	SE1.The nurse states to be convinced that she is able to offer the intervention map to the patient during the outpatient clinic visit.	A1.The nurse states to be convinced that he/she is able to offer patients the intervention map.	OE1.The nurse expects to improve patients’ knowledge and attitude when this intervention map is handed out to every patient.
Nurses actively invite patients to think about possible causes of their undernutrition.	K2.1.The nurse knows why it is important to let the patient think about the cause of undernutrition. K2.2. The nurse knows which factors may lead to undernutrition.	SE2.The nurse is convinced that she is able to actively invite the patient to discuss possible causes of undernutrition. SE2. The nurse is convinced that she is able to discuss possible causes of undernutrition.	A2.The nurse states that it is important to invite the patient to tell what a possible cause might be of undernutrition.	OE2. The nurse expects that the patient understand the personal causes of undernutrition.
Nurses inform and advice about the causes of undernutrition and energy- and protein rich food	K3. The nurse knows the causes and consequences of undernutrition and know the benefit of and what energy- and protein-rich food is.	SE3. The nurse states to be able to advise the patients about how to deal with underlying cause (s) and energy- and protein-rich food.	A3. The nurse is convinced that it is important to support the patient to have an good nutritional intake and status, and that she as a vital role in it.	OE3. The nurse expects that the patient know how to improve nutritional intake and status.
Nurses will instruct the patients to record nutritional intake in a food diary.	K4.1. The nurse knows the content of the food diary. K4.2. The nurse knows the procedure of recording the nutritional intake and how to help the patient with it.	SE4. The nurse states to be able to instruct the patient to record nutritional intake.	A4. The nurse states that it is important to instruct the patient to record nutritional intake.	OE4. The nurse expects the patient to be able to adequalty record his/her nutritional intake for 2 days.

One of the performance objectives for nurses state that nurses should inform and advise patients about the causes and consequences of undernutrition, about the need of energy- and protein-rich food, and about eating healthy snacks (see Table 4). The determinant knowledge requires nurses to be educated on these topics, and the determinant self-efficacy and skills requires nurses to be able to advise and encourage the patients on these topics. Regarding attitude, nurses need to be convinced of the need for nutritional care for surgical patients and of their important role in supporting patients in having an adequate nutritional status. Then, nurses should expect that the patient know how to improve his or her nutritional status and nutritional intake.

## Step 3: program design - methods

This phase of intervention development aims to identify theoretical methods which match with the determinants (step 1) and the program goals (step 2). Theories regarding undernutrition, methods of nutritional support, and behaviour change theories were considered relevant. These theories and methods were studied and discussed by the project group in order to conceptualise the intervention.

## Step 3: program design - results

Theories on the following subjects were selected: a) behaviour change [17, 22, 23]; b) undernutrition and nutritional care [4, 10, 11, 27, 32]; and c) implementation strategies [25, 26]. Table 5 displays the methods that were derived from these sources matching with patients’ and nurses’ determinants (see Table 5). These methods were applied in the conceptualisation of the program and taken into account in the program production during step 4 (see Table 5). The program focused on oral nutritional support for patients and training of the nursing staff. Key concepts of the behaviour change theory were applied to achieve the desired behaviour of both nurses and patients. These informed the structure of the support and the training. Key concepts from the sources on undernutrition and nutritional care were applied to define content of the support and the training. Key concepts from the implementation sources were applied to implement the support in nurses’ daily practise and to implement better nutritional behaviour in patients’ daily life.

**Table 5:** Application of methods per patient’ and nurse’ determinants

Determinant	Methods	Applications	How context and parameters were taken into account
Patients’ knowledge	Provide information using different methods about undernutrition and nutrition^f, g, i, j^	General and tailored information during the consults with advice and leaflets	Context: Consult during pre-operative evaluation and follow-up Parameters: patients received general information and advice orally by the nurse, received general leaflets. Questions were addressed and discussed.
	Increase memory and understanding^a^	Counselling during the consult and follow-up	
Patients’ awareness	Provide information about risks and consequences^a, f, g, i, j^ and encourage on desired behaviour^h^	General and tailored information during the consults with advice and leaflets	Context: Consult during pre-operative evaluation and follow-up Parameters: To tailor information, individual causes for undernutrition were determined and related advice was given; evaluation during follow-up to encourage the patient
	Tailor advices to the individual cause (s) of undernutrition^h^	Counselling during the consult and follow-up for encouragement and the nutritional care plan	
	Self-monitor nutritional intake^h^	Evaluation of the intake as recorded in a food diary during follow-up	Context: Consult during pre-operative evaluation and follow-up Parameters: by monitoring personal nutritional intake patients become aware
Patients’ skills	Instruct how to monitor nutritional intake^a, b, d^	Instruction of recording intake using a food diary during the consult	Context: Consult during pre-operative evaluation and follow-up Parameters: a food diary was supplied and patients were instructed to monitor intake.
	Instruct innovation of personal eating pattern^a, e, f^	Advice during the consult and evaluation during follow-up	
	Plan social support^h^	Follow-up by nurse or dietician	Context: Telephone follow-up Parameters: records of food intake were discussed and questions were addressed
Nurses’ knowledge	Refresh knowledge^c^ and provide information about behaviour-health link^a^, about undernutrition, its causes and consequencesWensing^g^, about nutrition during surgery^i, j^, and about behaviour change^12^	Training (given by dietician and nursing researcher) in which information is provided	Context: Training in small groups. Parameter: Schematic representations; an overview of current knowledge, adjusted to the knowledge level shown in individual interviews.
	Model or demonstrate the behaviour by modelling ^c^ Provide instruction by active learning, advance organisers, and cooperative learning^b^ Educational meetings by advance organisers, implementation intentions, and persuasive communication^c, d, e^	Training in which information is shown of the several steps of the intervention. Cases are discussed, and nurses did some role playing to exercise. Step-by-step written explanation of how the intervention must be carried out, given to nurses.	Context: Training in small groups. Parameters: a role play of the intervention during the training as an example and comparison with their own behaviour. Schematically displaying the intervention in the step-by-step written information. Discussing the ONNI^a^ during follow up meetings (once a week) to encourage nurses toward the adoption of the intervention.
Nurses’ self-efficacy and skills	Provide general encouragement, providing feedback on performance by mobilizing social support, consciousness raising and feedback^a, b, c^	Nurses give feedback to the researcher during role play, and the researcher visits the outpatient clinic to discuss feedback.	Context: the nursing teams at the outpatient clinics included are relatively small and therefore easily approachable, and visiting the outpatient clinic is a low-key approach in talking to the nurses. Parameters: Specific feedback is given, nurses are given the opportunity to talk about the use of the ONNI, and their behaviour, encouraged by the researcher.
	Prompt barrier identification and reviewing practice and feedback by planning coping responses and discussion^a^	Individual interviews in which nurses are invited to think about barriers and facilitators around the nursing nutrition intervention, and weekly meetings in which the use of the intervention is discussed.	Context: All nurses of the outpatient clinic were interviewed. Usual care was observed, in both hospitals. Parameters: While designing the intervention, potential barriers, based on observations and interviews, were identified and the expert team discussed on what was needed to overcome these barriers.
	Provide information about colleagues’ approval by modelling and information about others’ approval^c^ Stimulate discussion between nurses by mobilizing social support and guided practice^a, c^	Follow-up meetings with nurses in the intervention groups (answering questions, discussing experiences)	Context: Weekly follow-up meetings with nurses Parameters: discuss cases, what went well and what could be improved; intervention performance with positive aspects and challenges.
Nurses’ attitude	Provide information about patients’ perspective by shifting perspective ^e^ Provide overview of the nursing role in (under) nutrition^a, h^ Validate and empower on desired behaviour^c^ Visits to the outpatient clinics by researchers^a, b, c, d^	Training and follow-up meetings in which quotes from patients are discussed.	Context: Training in small groups and weekly follow-up meetings with nurses. Parameters: Quotes from observations of usual care and the nursing nutrition intervention were discussed to encourage nurses to take the perspective of the patient to increase the adoption.

^a^Outpatient Nursing Nutritional Intervention; ^a^Abraham et al., 2008 [22]; ^b^Van Achterberg et al., 2011 [23]; ^c^Grol & Grimshaw, 2003 [24]; ^d^Grol et al., 2007 [25]; ^e^Wensing et al., 2010 [26]; ^f^Daniels et al., 2003; Jensen et al., 2009; www.fightmalnutrition.eu; ^g^Weimann et al., 2017 [10]; ^h^Van Noort et al., 2019 [11]; ^i^McClave et al., 2013; ^j^West et al., 2017 [27]

The way we applied the theories in development of the intervention is explained in the following example: In step 1, it turned out that most of the patients were unaware of undernutrition and its consequences. When the researcher informed the patient adequately during the interview, some stated that they felt to be able to maintain their weight. Therefore, we considered awareness, attitude, self-efficacy and skills as important determinants to be changed. According to the program goal for patients (to improve or maintain nutritional status), performance objectives stated that patients need to take action regarding their individual cause (s) of undernutrition and eat healthy, energy and protein enriched nutrition (see Table 3). Theories on behaviour change techniques [22, 23] argue that healthy behaviour can be obtained through social support and self-monitoring. Also, components of oral nutritional support included counselling at several points in time [11]. Therefore, the project group argued that patients should be encouraged and counselled at several points before hospital admission by both caregivers and healthcare professionals. Encouragement (e.g. social support) and counselling were scheduled two times before surgery, i.e., during the consults at the anaesthesia outpatient clinic and during a follow-up telephone call within a week after the consult. During the consultation, nurses can inform, empower and support the patients and actively involve the caregivers during the consult.

## Step 4: program production - methods

The project group synthesised the information from previous steps to determine the program consisting of an Outpatient Nursing Nutritional Intervention and a nursing nutritional training. One researcher (GHdW) prepared all versions of the intervention and presented these for comments to the rest of the project group. After three rounds of feedback, consensus was reached.

The training for the outpatient-clinic nursing staff was developed to help the nurses achieve their change objectives. A researcher (GHdW) and the dietician (MvA) of the project group developed the training using the methods and applications mentioned in Table 5.

Two nurses of the outpatient clinic of the academic hospital tested the ONNI after the training in six consults to evaluate if the ONNI could work [33]. Both nurses participated in the interviews of step 1 after written informed consent. Before the training they were unaware of the importance of nutritional status for patients outcomes. The nurses perceived that the nursing role was limited to nutritional screening and did not know how they could provide nutritional care.

The ONNI and the training were evaluated using a short questionnaire and interviews. Topics covered in the semi-structured questionnaire concerned the experiences of nurses with the training and the extent of improvement on the previously identified determinants as a result of the training. The interview based on this semi-structured questionnaire was held in person with a researcher. The six consecutive patients who received the ONNI were interviewed after written informed consent. Objective was to determine the extent to which patients were exposed to different intervention components during the consults, patients’ ability to record food intake, and their awareness of nutrition and eating patterns. Patients were also questioned about their preferences regarding two types of food diaries. Notes were made after each interview and analysed through open coding.

## Step 4: program production - results

### The outpatient nursing nutrition intervention

The ONNI was developed for use during outpatient pre-operative consults and consists of five components (see Table 6). First, causes of undernutrition were determined with a checklist. Then, a nutritional care plan aimed to educate the patient with both tailored and general information. In case of a MUST score ≥ 2, patients were also referred to the dietician (usual care). The third component aimed at providing insight in patient’s eating pattern by recording daily intake for 2 days in a food diary. The fourth component was to counsel and encourage the patient in improving nutritional status during the outpatient clinic visit and a follow-up meeting. The fifth component was support during a telephone follow-up meeting with the patient within 1 week after the outpatient clinic visit. The ONNI was targeted at patients at risk for or with undernutrition based on MUST scores.

**Table 6:** The five components of the Outpatient Nursing Nutritional Intervention (ONNI)

Component	Content
1) Determine causes of undernutrition	Possible causes of undernutrition were: a) bad appetite, b) decreased intake, c) gastrointestinal problems, d) insufficient physical activity, e) pain, or f) poor oral health
2) Perform a nutritional care plan	A: provide tailored advice related to possible cause (s) B: provide leaflets on ‘energy and protein enriched nutrition’^a^ and ‘tempting food’ C: refer the patient to the dietician in case of MUST score ≥ 2^a^
3) Self-monitoring of nutrient intake and eating pattern	A: explain the patient how the food diary works and how to record daily intake within the diary B: instruct the patient to monitor food intake for 2 days in the dairy
4) Counselling and encouragement	A: counsel the patient on eating patterns and encourage the patient to improve nutrient intake B: advice the patient to inform caregivers and/or involve caregivers during the consult C: plan a telephone follow-up meeting with the patient to be held after approximately 1 week
5) Follow- up meeting^b^	A: evaluate how causes of undernutrition did work out B: evaluate the food diary on total intake and the nutrients that were consumed C: counsel and provide tailored advice on energy and protein enrich products and on causes of undernutrition

^a^activities of usual care, and was therefore included in the ONNI; ^b^performed by the nurse of the outpatient clinic or, in case of MUST score ≥ 2, by the dietician

### Training

The nursing nutritional training consisted of three plenary meetings. Two of the three meetings were aimed at increasing nurses’ knowledge of undernutrition, its causes and consequences, behaviour, and health, along with information about the intervention protocol. Also, to raise awareness the role of nurses in meeting patients’ needs including nutritional needs was elaborated during this training through providing an overview of the nurses’ role in undernutrition. To increase their skills and self-efficacy, nurses practised the intervention in a role play during the meeting to see examples and make comparisons to their own behaviour. Additionally, to increase their self-efficacy and improve their attitudes towards their nutritional roles, interactive discussions exploring nurses’ individual perspectives were held during the training. Nurses discussed how to deal with the patients’ points of view using personal experiences. These discussions helped to set a peer group and determine social norms. The third meeting aimed to clarify the intervention protocol and to invite nurses to explain the steps of the intervention to receive feedback from the trainer. Follow-up meetings at the outpatient clinic were scheduled with the trainer and nurses to deal with remarks or queries.

Changing the attitude towards positive awareness of nurses’ role in nutritional care was addressed during the training sessions and follow-up meetings. Increasing knowledge, exploring individual and patients’ perspectives, several discussion sessions on different time points, and performing the intervention during training sessions and in daily practise will together lead to the desired behaviour. The nursing staff include 10 nurses in total. For the evaluation in step 6, nurses will be randomised to perform the ONNI or usual care. Therefore, attitude of five nurses are to be changed. The researcher is therefore able to coach nurses individually which would lead to optimal attitude and intervention delivery.

### Test

The nurses (*N* = 2) stated that the training refreshed and updated their knowledge regarding undernutrition and that information on the intervention was clearly provided. They showed willingness to meet patients’ nutritional needs and felt responsible to improve patients’ nutritional status. The nurses felt that the intervention was complete and applicable in practice. After completing the intervention for three patients, the nurses stated that they were able to perform the full intervention adequately. They also stated that they were able to carry out the intervention in the time allocated for each patient and that they became more familiar with the ONNI.

Patients (*N* = 6) stated that they received all the information necessary, were able to use the food diaries, and became aware of their eating patterns by using the diaries. The patients used both the hospital food diary and the Dutch Malnutrition Steering Group food diary. After evaluation, all patients preferred the hospital version of the food diary (see Additional file 4).

## Step 5: program implementation plan - methods

This step involves the adoption and implementation of the ONNI in daily practise. The intention was that the ONNI should be used in the two anaesthesia outpatient clinics to allow for an evaluation of the feasibility and effectiveness of the intervention.

Literature on effective implementation strategies [34, 35] and methods to evaluate complex interventions in health care [24–26] were used to determine the implementation plan. First, desired behaviours for patients and nurses were derived from the previous steps. Then, barriers to performance of the desired behaviours and adherence to the program goals (step 3) were identified based on observations in current practices, interviews with nurses and patients (step 1), and questionnaires completed by patients (step 4). Finally, implementation strategies from the literature were matched with these barriers and desired behaviours.

## Step 5: program implementation plan - results

The determinants and barriers identified in previous steps required a multifaceted implementation strategy [24–26]. The project group considered a) lack of awareness of their responsibilities in nutritional care, b) lack of prioritisation during consults, and c) the feeling of being unable to provide nutritional care for undernourished patients as the most important barriers for nurses to adapt and implement the desired behaviour. Lack of knowledge about undernutrition and interventions was also a barrier, however, the training was considered to adequately elevate the nurses’ knowledge. For patients, an expected challenge was recording food intake in a food diary. These barriers required a multifaceted implementation strategy and included education, evaluation of the education, feedback during performance for nurses, and evaluation of the types of food diaries for patients (see Table 7).

**Table 7:** Implementation strategy for adaptation and use of Outpatient Nursing Nutritional Intervention (ONNI)

Implementation strategy	Users	Content	Professionals involved
Education	Nurses	What: Relevant training sessions with regard to disease-related undernutrition and the intervention protocol When: 1 month before the start of the intervention period How: Two interactive meetings about the basic principles of the intervention protocol	Dietician, researcher, and nurses
Evaluation of the training	Nurses	What: discussion about intervention protocol When: 1 week after the training How: clarifying by the dietician and researcher, explaining of the ONNI steps by nurses, feedback for nurses about their performance on ONNI	Dietician, researcher, and nurses
Feedback	Nurses	What: Feedback on nurses’ performance When: During implementation How: Observation at the outpatient clinic by a researcher	Dietician, researcher, and nurses
Evaluation of type of food diary	Patients	What: Evaluation of patients’ preferences for food diary When: During test of the ONNI How: Providing two types of food diaries	Researchers, nurses

The researcher (GHdW) observed the way nurses performed the intervention. Afterwards, the researcher and the nurse discussed the performance and the researcher gave feedback to the nurse. Also, the nurses’ experiences with the intervention were discussed with the researcher who visited the outpatient clinic during weekly follow-up meetings in the implementation period. These discussions were meant to increase nurses’ skills and improve their attitudes towards nutritional support. Nurses asked the trainer more questions during the first meetings compared to the end of this period. Near the end of the implementation period, nurses started to feel familiar with the intervention.

## Step 6: evaluation plan

In the final step, the aim is the design of an evaluation study, which required an evaluation of the feasibility and effectiveness of the ONNI. This evaluation is not the focus of this paper and is reported separately [36]. The study protocol was registered at the National Institutes of Health (NIH) with the ClinicalTrial.gov Identifier, NCT02440165 [37].

## Discussion

This paper describes the methods and end products of the application of the IM approach to develop an ONNI.

Within the MRC framework for complex interventions, IM was used to structure the development phase of the complex nutritional intervention. In step 1, research identified the determinants which contributed to undernutrition or risk for undernutrition before surgery. In this phase of development, we invited patients to share their opinions and experiences. The stakeholders collaborating in the project group all had experience with nursing, undernourished patients, systematic development of interventions, or held a combination of these areas of expertise. Patients were not included in the project group. Involvement of patients was addressed in step 1 by exploring their perspectives, and also during evaluation of the intervention in step 4. Involvement of patients’ perspectives is considered as ‘consulting’ on the ladder of citizen participation [38]. Partnership of patients in the design of the study and the intervention would require expert contribution from the patient which appeared difficult according to previous intentions [39]. Therefore, we preferred active patient participation in their nutritional prehabilitation rather than their partnership in the design of this study. We argue that we had a strong theoretical framework, recent evidence, and clinical expertise to thoroughly develop an evidence-based intervention. Additionally, the intervention is tailored to the identified barriers to change and behaviour which is recommended to achieve improvements in professional practice [34].

With regard to the entire process of intervention development, we argue that although IM is time consuming, it results in an examination of the context, an evidence-based, thought-through intervention, as well as training and a set of implementation strategies. At this time, a test confirmed an improvement in nurses’ behaviour and patients’ knowledge and skills. However, the feasibility and effectiveness of the ONNI are not yet determined. The evaluation plan, step 6 of IM, is meant to determine the feasibility of the ONNI in daily practice and the effectiveness effect based on relevant nutritional outcomes [37]. This examination of the ONNI in daily practice will optimize the development phase [40].

Nurses were found to be unaware of their roles and felt incapable of providing nutritional care (step 1). This is in accordance with other studies, which demonstrated that nurses were unaware of their nutritional roles, demonstrated low self-efficacy, and lacked nutritional knowledge [41, 42] and education [43, 44]. Since nutrition belongs to the core of basic nursing care [28, 45, 46], nurses need to be educated on their crucial roles regarding nutrition to ensure that patients are properly fed. The training (step 4) has the potential to address this challenge. In our test, we found that nurses’ attitude changed as they became aware of their role in nutritional care. As the nursing staff was small, a crucial instrument would be that the researcher can supervise each nurse individually during the implementation at the outpatient clinic.

In the samples of surgical outpatients (step 1), 5–7% of the patients were at risk for undernutrition, and 4–5% of patients were undernourished (see Table 2). Despite the fact that these rates are in accordance with other studies in this setting [5, 7], the literature demonstrates higher percentages of patients at risk for or with undernutrition among other populations [4, 47–49]. The samples in our study may have been healthier and younger, and patients in our sample were only seen for surgery. The ONNI can be adapted to all types of outpatient clinics, since undernutrition is seen in all types of medical specialties [16, 49]. The fact that the ONNI was especially developed for preoperative outpatients must be taken into account. Researchers and policymakers can use several intervention frameworks [17, 50, 51] for further adaption of the ONNI.

Moreover, as a result of the survey during the needs assessment, well-nourished patients stated that they needed additional information regarding nutrition (step 1). This may suggest that patients are generally not well informed regarding nutrition and that all patients may benefit from nutritional advise during outpatient preoperative consultation.

The ONNI comprises tailored and general advice, leaflets, counselling on eating patterns based on a food diary, encouragement for sufficient and healthy food intake, and a follow-up meeting. It is intended to be provided in anaesthesia outpatient clinics before patients’ admission for surgery. Other oral nutritional interventions include education of caregivers of dependent undernourished patients [52], enhanced recovery protocols during hospital admission [27, 53, 54], and dietetic consultations starting at hospital discharge with fortnightly follow-up meetings for 6 months [55]. This ONNI focuses on nutritional support for the patient but involves caregivers only during the consultation at the outpatient clinic. Further research should address family participation during surgical course. Additionally, further research should address nutritional support during the whole surgical course, i.e. preoperative and postoperative phases. Researchers and health care professionals can adapt the ONNI components and evaluate these during hospital admission and after hospital discharge.

Surgical patients with undernutrition may also have other frailty factors that increase the risk of complications. Levett, Edwards, Grocott, and Mythen (2016) discuss that preoperative prehabilitation of high-risk patients should be based on a multimodal and interdisciplinary approach [56]. The health status of patients at risk for or with frailty should be optimized through preoperative physical, nutritional, and psychological optimization. Our intervention contributes to the nutritional prehabilitation, and our study provides the definition of the nurses’ role within the inter-professional approach [57].

## Conclusions

This application of the IM approach demonstrates that nurses at the outpatient clinics felt incapable and did not feel responsible for delivery of preoperative nutritional support to surgical patients at risk for or with undernutrition. Patients themselves were often unaware of their nutritional status and the increased risks for complications in case of undernutrition. The extensive IM approach resulted in an evidence-based, thoroughly-developed ONNI. The ONNI, including a training for nurses, aims to improve or maintain patients’ nutritional status. The test confirmed improved knowledge, skills, and sense of responsibility in nurses. The ONNI enables nurses to empower patients to improve their preadmission nutritional status and ultimately may improve postoperative recovery.

## Supplementary information

**Additional file 1.** Interview guide to explore nurses’ perspectives.

**Additional file 2.** Survey on patients’ satisfaction with general and nutritional care.

**Additional file 3.** Interview guide for patients’ perspectives.

**Additional file 4.** Food Diary.

## Data Availability

The data generated and analysed during the current study are not publicly available but are available from the corresponding author upon reasonable request. The materials used and developed during the study are provided as supplementary materials.

## Abbreviations

EN: Enteral Nutrition
IM: Intervention Mapping
MRC: Medical Research Council
MUST: Malnutrition Universal Screening Tool
ONNI: Outpatient Nursing Nutritional Intervention
ONS: Oral Nutritional Supplements
PN: Parenteral Nutrition
