# Supplementary material for: Using intervention mapping to develop an outpatient nursing nutritional intervention to improve nutritional status in undernourished patients planned for surgery
Source: BMC Health Serv Res. 2020 Feb 27;20:152. doi: 10.1186/s12913-020-4964-6 (PMC7047387; doi:10.1186/s12913-020-4964-6)
Supplement: Supplementary file 1 — Additional file 1. Interview guide to explore nurses’ perspectives. [file 12913_2020_4964_MOESM1_ESM.docx]

# Additional file 1 - Interview guide to explore nurses’ perspectives

1. In your opinion, is nutrition part of your profession as a nurse? If so/if not, can you explain why? *This question helps find out about the attitude of the nurses and determine whether they see nutrition as their responsibility.*
2. Do you feel responsible for providing nutritional advice as a nurse? If so/if not, can you explain why? *This is an additional and more detailed question than question 1.*
3. Can you explain what undernutrition is, how it is caused, and what it may cause? *This question detects the nurses’ level of knowledge regarding undernutrition.*
4. Do you think you have sufficient knowledge of undernutrition? *This question determines nurses’ effectiveness and self-efficacy.*
5. How did you obtain this knowledge? *This question helps detect awareness and recourses for information and knowledge.*
6. Nutrition is at the heart of nursing. To what extent do you agree with this statement?

□ Strongly agree

□ Agree

□ Not agree/not disagree

□ Disagree

□ Strongly disagree

*This question/statement elicits nurses’ attitudes towards nutrition.*

1. Can you explain why it is important for preoperative patients to consume a sufficient amount of energy and protein? *This question helps detect awareness and knowledge.*
2. Are there any questions from patients regarding nutrition that you cannot answer adequately during consults? If so, what are those questions and what do you do? *This question detects nurses’ effectiveness and self-efficacy.*
3. Are you up to date regarding innovations in nutrition? If so, how do you stay up to date? *This question detects nurses’ recourses for information and knowledge and their attitude for staying up to date.*
4. I screen for undernutrition because I know it is important for the patient to have an optimal nutritional status before surgery. To what extent do you agree with this statement?

□ Strongly agree

□ Agree

□ Not agree/not disagree

□ Disagree

□ Strongly disagree

*The argumentation for this statement illustrates nurses’ attitude, awareness, and knowledge.*

1. Are you confident in providing information to a patient regarding nutrition? *This questions concerns self-efficacy.*
2. My colleagues encourage me to screen for undernutrition using MUST. To what extent do you agree with this statement?

□ Strongly agree

□ Agree

□ Not agree/not disagree

□ Disagree

□ Strongly disagree

*The argumentation for this statement indicates nurses’ social influences.*

1. How would you feel if you as nurse were to play a major role in advising patients during consults? *This question elicits the nurses’ attitude.*
2. What topics are discussed during consults with patients? *This question uncovers nurses’ current behaviours during consults.*
3. Are all the tools necessary for measuring patients’ nutritional status available at the outpatient clinic? *This question elicits nurses’ skills and awareness.*
4. Do you measure the height and weight of patients? *This question helps find out what the components of the intervention can be. It determines the awareness and self-efficacy of the nurses.*
5. What activities do you perform in case of a MUST score of 1? What do you do in the case of a MUST score of at least 2? *This question detects current performance and skills.*
6. Are there any challenges for you when advising the patient on nutritional behaviour? *This question identifies the potential barriers to developing the intervention.*
7. What do you prefer with regard to the content of the intervention to be developed? What elements of an intervention can you benefit from? *With this question, nurses’ opinions can be used in the development of the intervention.*
