# Supplementary material for: Using intervention mapping to develop an outpatient nursing nutritional intervention to improve nutritional status in undernourished patients planned for surgery
Source: BMC Health Serv Res. 2020 Feb 27;20:152. doi: 10.1186/s12913-020-4964-6 (PMC7047387; doi:10.1186/s12913-020-4964-6)
Supplement: Supplementary file 2 — Additional file 2. Survey on patients’ satisfaction with general and nutritional care. [file 12913_2020_4964_MOESM2_ESM.docx]

# Additional file 2 – Survey on patients’ satisfaction with general and nutritional care

| 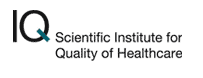  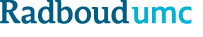   What is your opinion?  *Questionnaire on your experience at the outpatient clinic of the hospital*  Only for persons of at least 18 years of age.  **<patient identification sticker>**  This questionnaire will take about 10 minutes to complete. |
| --- |

**Confidential**

- All information will be handled confidentially.
- Personal data will not be shared without permission.
- The hospital staff and health insurer will not see your personal answers.
- We ensure that all questionnaires are kept anonymous.
- A patient sticker and/or file number is on the questionnaire. We use this information to know whether you have returned the questionnaire or not. This way, we know we do not have to remind you.

**VOLUNTARY PARTICIPATION**

- We kindly ask if you would like to complete this questionnaire.
- Participation in this study is voluntary. Whether or not you participate does not impact any medical treatment.

**QUESTIONAIRE INSTRUCTIONS**

- This questionnaire concerns your visit to the nurse/nursing assistant at the anaesthesia outpatient clinic of <hospital name>. Please do not include visits to other outpatient clinics in your answers.
- It is important for our research that you answer all the questions and do not skip any questions.
- When you want to change an answer, please use parenthesis to identify the wrong answer and use a cross for the right answer, as illustrated below:

(🗵) Yes

🗵 No**General questions**

The following questions are general questions about you. We use the information to gain insight in experiences of different groups of people.

1. Are you male or female?

❑ Male

❑ Female

2. What is your age? …………

3. What is the highest level of education you have completed?

❑ No education

❑ Primary education

❑ Secondary education

❑ Vocational education

❑ University of applied sciences

❑ Academic education (university)

4. What is your country of birth?

❑ The Netherlands

❑ Other:……………………………

5. How would you qualify your general health?

❑ Excellent

❑ Very good

❑ Adequate

❑ Moderate

❑ Bad

**CARE OF THE NURSE OR NURSING ASSISTANT DURING OUTPATIENT CLINIC VISIT**

6. Did the nurse / nursing assistant take you seriously?

- No, not at all
- A little
- Mostly
- Yes, completely

7. Did the nurse / nursing assistant listen to you carefully?

- No, not at all
- A little
- Mostly
- Yes, completely

8. Did the nurse / nursing assistant take enough time for you?

- No, not at all
- A little
- Mostly
- Yes, completely

9. Was the nurse / nursing assistant an expert?

- No, not at all
- A little
- Mostly
- Yes, completely

10. Did the nurse / nursing assistant discuss the importance of nutrition before surgery and hospitalization with you?

- Yes
- No

11. If so, what topics were addressed? (multiple answers possible)

- Weight and height were measured
- Received information on undernutrition
- Received information on good nutrition before surgery
- Received information on complications after surgery due to undernutrition
- Received advice on energy- and/or protein-rich products
- Received advice on the use of extra mealtimes
- Other: ______________________________________________________________________________________________________________________________________

12. If not, what information would you like to receive: (multiple answers possible)

- Information on undernutrition
- Information on good nutrition before surgery
- Information on complications after surgery due to undernutrition
- Advice on energy- and/or protein-rich products
- Advice on the use of extra mealtimes
- Other:
  ___________________________________________________________________

13. How would you rate the care of the nursing care **regarding nutrition** at the outpatient clinic?

0 indicate: very bad; a 10 indicate: excellent

- 0
- 1
- 2
- 3
- 4
- 5
- 6
- 7
- 8
- 9
- 10

**OVERALL CARE**

14. How would you rate the care of the nurse / nursing assistant at the outpatient clinic?

0 indicate: very bad; a 10 indicate: excellent

- 0
- 1
- 2
- 3
- 4
- 5
- 6
- 7
- 8
- 9
- 10

15. How would you rate the outpatient clinic?

0 indicate: very bad; a 10 indicate: excellent

- 0
- 1
- 2
- 3
- 4
- 5
- 6
- 7
- 8
- 9
- 10

16. Would you recommend the outpatient clinic to your family and friends?

- Definitely not
- Probably not
- Probably
- Definitely

**GENERAL HEALTH**

17. How would you rate your appetite over the last week?

0 indicates ‘I had no appetite at all’ and 10 indicates ‘my appetite was very good’.

- 0
- 1
- 2
- 3
- 4
- 5
- 6
- 7
- 8
- 9
- 10

18. How would you rate your nutritional status in general?

- Excellent
- Very good
- Adequate
- Moderate
- Bad

19. What is the importance of protein in your nutrition?

- Proteins are not important.
- Proteins are vital to build my body and are important for both muscles and wound healing.
- Proteins are important for wound healing only.

20. Are you motivated to prepare for surgery by consuming protein-rich nutrition?

- Very motivated
- Somewhat motivated
- Not motivated

21. Have you been referred to a dietician?

- Yes
- No

22. Do you have sufficient financial resources to adapt your grocery shopping to a healthier eating pattern?

- Definitely not
- Probably not
- Probably
- Definitely

**GENERAL HEALTH**For each group below, draw a cross on the box to indicate the statement that best reflects your health today.

MOBILITY

I have no trouble walking. ❑

I have some trouble walking. ❑

I have serious trouble walking. ❑

I have very serious trouble walking. ❑

I am not able to walk ❑

PERSONAL HYGIENE

I have no trouble cleaning and dressing myself. ❑

I have some trouble to clean and dress myself ❑

I have serious trouble to clean and dress myself ❑

I have very serious trouble to clean and dress myself ❑

I am not able to clean and dress myself ❑

DAILY ACITIVITIES

(for example: work, study, housekeeping, family and leisure activities)

I have no trouble with daily activities ❑

I have some trouble with daily activities ❑

I have serious trouble with daily activities ❑

I have very serious trouble with daily activities ❑

I am not able with daily activities ❑

PAIN/DISCOMFORT

I have no pain or discomfort ❑

I have some pain or discomfort ❑

I have serious pain or discomfort ❑

I have very serious pain or discomfort ❑

I have extreme pain or discomfort ❑

ANXIETY/DEPRESSIVE FEELINGS

I have no fear or sad feelings ❑

I feel a little anxious or sad ❑

I feel anxious or sad ❑

I feel very anxious or sad ❑

I feel extremely anxious or sad ❑

We ask how good or bad your health is TODAY.

10

0

20

30

40

50

60

80

70

90

100

5

15

25

35

45

55

75

65

85

95

**The best health you can imagine**

**The worst health you can imagine**

This scale ranges from zero (0) to one hundred (100).

100 indicates the best health you can imagine.
0 indicate the worst health you can imagine.

Mark an X on the scale to indicate your health status today.

Mark the number next to your X in the box below.

YOUR HEALTH STATUS TODAY =

**Space for comments**

Thank you very much for completing this questionnaire!

You can hand this to the nurse/nursing assistant.
