# Supplementary material for: Using intervention mapping to develop an outpatient nursing nutritional intervention to improve nutritional status in undernourished patients planned for surgery
Source: BMC Health Serv Res. 2020 Feb 27;20:152. doi: 10.1186/s12913-020-4964-6 (PMC7047387; doi:10.1186/s12913-020-4964-6)
Supplement: Supplementary file 3 — Additional file 3. Interview guide for patients’ perspectives. [file 12913_2020_4964_MOESM3_ESM.docx]

# Additional file 3 – Interview guide for patients’ perspectives

**Introduction**

We are interested in your perspective on your nutritional status, on your contribution to improving or maintaining your nutritional status and on the nutritional care you may have received during outpatient clinic consultation.

The interview consists of open-ended questions. The questions concern nutrition and food. If necessary, we will ask other questions for more information based on your answers. This interview will be recorded, if you consent. Please feel free to let us know if you have any doubts or questions before we start. The recording will be transcribed on paper, and your information will be kept anonymous. The interview will take about 20 to 45 minutes. Do you provide your consent for this interview? Do you have any questions?

**Patient characteristics**

1. What is your nationality?
2. What is your marital status?
3. What is your living situation?
4. What is the highest level of education you have completed?

**Topics:**

**Knowledge and attitudes regarding undernutrition and nutritional intake**

1. Do you know that you were screened for undernutrition during your visit to the anesthesia outpatient clinic? Do you know that the screening determined that you are undernourished or at risk of becoming undernourished?
   1. Possible explanation: this may be because you weigh less than normal according to your height or because you have lost some weight unintentionally.
2. Can you explain undernutrition and risk for undernutrition?
3. Are you already familiar with undernutrition or what it means to be at risk for undernutrition?
   1. If so, have you been supported by health care professionals?
4. What is your opinion about the fact that you have been labeled as being undernourished or being at risk for undernutrition?

**Patients’ experiences with received nutritional care**

1. Can you talk about the care you received to improve your nutritional status?
2. Did the nurse help you decrease your level of undernutrition or the risk for undernutrition during the outpatient clinic visit?
   1. If so, what actions did the nurse take?
   2. If not, did you receive verbal or written information on nutrition?
3. **[in case of MUST-score 2 (undernutrition)]**Have you been referred to a dietician?
   1. If so, did you actually see the dietician?

**Patients’ needs and expectations regarding nutritional care**

1. Do you need other support from the caregivers? Was anything lacking in the actions of the caregiver at the outpatient clinic?
2. Can you talk about your expectations regarding the treatment for undernutrition or the risk for undernutrition?

**Responsibilities and motivations regarding undernutrition and nutritional intake**

1. What do you do yourself to improve your nutritional status?
2. Can you explain your opinion on your personal responsibility for improving nutritional status? What do you think you can do yourself to diminish nutritional risk?
3. Did the nurse provide you with any specific recommendations to improve your nutritional status yourself?
   1. If so, what were these recommendations, and did you comprehend these recommendations?
   2. Did you apply these recommendations after the outpatient clinic visit? Why?
   3. Were the recommendations feasible for you?
   4. How did you think about applying the recommendations at home?
4. What makes it easier for you to work to improve or maintain your nutritional status?
5. What makes it harder for you to work to improve or maintain your nutritional status?
6. Can you go to someone when you face troubles with your nutritional status?
7. Do you think you would go if it was necessary?

**Disclosure**

This is the end of the interview. Do you have any questions or remarks in response to this conversation?

Thank you very much for your co-operation!
