# Supplementary material for: Using intervention mapping to develop an outpatient nursing nutritional intervention to improve nutritional status in undernourished patients planned for surgery
Source: BMC Health Serv Res. 2020 Feb 27;20:152. doi: 10.1186/s12913-020-4964-6 (PMC7047387; doi:10.1186/s12913-020-4964-6)
Supplement: Supplementary file 4 — Additional file 4. Food Diary. [file 12913_2020_4964_MOESM4_ESM.docx]

# Additional file 4 – Food Diary


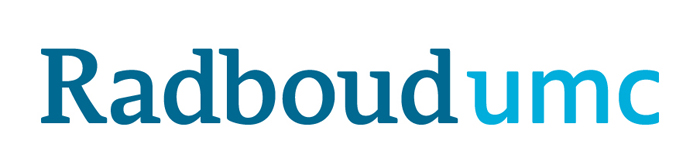


|  |  |
| --- | --- |

Encouter risk for undernutrition

Introduction

*This leaflet provides information for you to fill in the food diary. In the next period, it is important to maintain or obtain an adequate physical condition. For this, you need sufficient amounts of healthy nutrients.*

With this food diary, we try to gain insight into your eating patterns. Based on what you write, we can estimate your daily food intake.

To evaluate your food intake, the nurse will call you by telephone in about one week. She will discuss the food diary with you.

The follow-up telephone call will be at the following time:

Date Time

_______________________ ____________hour

Food Diary Instructions

The food diary needs to be completed for two days: one day during the week and one day during the weekend. It is necessary that you write everything you eat and drink in the diary. If possible, try to fill in the diary right after you eat or drink something.

Sizes and weights

You can write foods in household sizes and weights as follows:

| Foods (category) | Household size/weight  Amount in .. |
| --- | --- |
| Bread | A slice, sandwich, baguette |
| Meats | slices |
| Cheese | Slices (1 slide pre-cut = on average 20 grams) |
| Butter / margarine | Thin, normal or thickly smeared |
| Drinks | Glasses, cups, or mugs |
| Soup | Plates, bowls or mugs |
| Potatoes | Pieces of the size of 1 egg |
| Vegetable | Serving Spoons |
| Rice, pasta, fries, baked potato | Serving spoons |
| Meat / fish | Grams / serving (1 serving = average 100 grams) |
| Gravy and sauce | Gravy-, sauce- or tablespoon |
| Dessert | plate or dish |
| Chips / pretzels | Hands, dishes, bags |
| Cake / candy / chocolate | Pieces |
| Fruit | Pieces |

Example

| Eating and drinking | Amount |
| --- | --- |
| Breakfast |  |
| Soft white bun  Margarine  Jam and 30+ cheese  Coffee with cup of coffee milk | 2 pieces  Normally coated  Pre-cut slice of cheese  1 mug |
| During the morning |  |
| 1 glass water  Evergreen | 200 ml  1 glass |
| Lunch |  |
| Brown bread  Margarine  sausage and salami  peanut butter  Semi-skimmed milk | 3 slices  Normally coated  Both sliced 2 slices  for 1 slice  1 mug |
| During the afternoon |  |
| 2 glasses juice  apple | 2x 200 ml  1 piece |
| Diner |  |
| Boiled potatoes  Cooked green beans  Cordon bleu  Gravy from a package  Vanilla custard | 3 pieces 3 serving spoons 1 piece. Baked in 3tbspliquid butter 1 spoon 1 dish |
| During the evening |  |
| Chips  2 glasses Coca Cola light | 1 small bag  2x 200 ml |

Day 1

| Eating and drinking | Amount |
| --- | --- |
| Breakfast |  |
|  |  |
| During the morning |  |
|  |  |
| Lunch |  |
|  |  |
| During the afternoon |  |
|  |  |
| Diner |  |
|  |  |
| During the evening |  |
|  |  |

Day 2

| Eating and drinking | Amount |
| --- | --- |
| Breakfast |  |
|  |  |
| During the morning |  |
|  |  |
| Lunch |  |
|  |  |
| During the afternoon |  |
|  |  |
| Diner |  |
|  |  |
| During the evening |  |
|  |  |
